# 7, 8-Dihydroxyflavone Protects an Endothelial Cell Line from H_2_O_2_ Damage

**DOI:** 10.1371/journal.pone.0135345

**Published:** 2015-08-12

**Authors:** Bingxiang Wang, Qian Zhang, Ruyong Yao, Xiangping Liu, Zhiqiang Qu

**Affiliations:** 1 Center for Medical Research, the First Affiliated Hospital, Qingdao University, Qingdao, 266555, China; 2 Department of Physiology, Taishan Medical College, Taian, 271000, China; University of Sassari, ITALY

## Abstract

7, 8-dihydroxyflavone (7, 8-DHF), a selective agonist for TrkB receptors, has been well studied for its neurotrophic functions. However, its roles outside the neural tissues have scarcely been studied as yet. In this study, we investigated the protecting roles of 7, 8-DHF in EA.hy926 cells, a human umbilic vein endothelial cell line which was exposed to hydrogen peroxide (H_2_O_2_). We found that 7, 8-DHF significantly protected the cells from being damaged by H_2_O_2_ through suppression of apoptosis, attenuation of inflammatory factor releasing and inhibition of reactive oxygen species generation. The potent biological effects of 7, 8-DHF were probably executed via its binding to TrkB receptors because the receptor specific antagonist ANA-12 significantly blocked its protecting effects. The protecting roles of 7, 8-DHF in EA.hy926 cells suggest that it will be a promising compound to be developed into a health product that definitely benefits endothelial functions and prevents cardiovascular diseases.

## Introduction

7, 8-dihydroxyflavone (7, 8-DHF), a kind of flavonoids which are rich in fruits and vegetables, has been well demonstrated to be a selective small molecule agonist for TrkB receptors and possess strong neuroprotective function[1]. In order to explore its more applicable roles, we previously studied its effect on blood vessels and revealed that 7, 8-DHF was a potent artery-relaxing compound[2]. Interestingly, the relaxing effect was partially dependent on the intact endothelia and mediated by endothelial NO synthesis. We also confirmed its strong antioxidant effect in PC 12 cells through PI3K/Akt pathway[3, 4]. The results have invited us to hypothesize that 7, 8-DHF was probably able to affect or regulate other functions of the endothelia as well and might play a role in endothelial protection.

The vascular endothelia expose itself to blood, sensing directly the changes of the components in the blood. Because of its anatomical localization lining the smooth muscle layer, it has been well known that dysfunction of vascular endothelia is the first step towards the development of vascular diseases, e.g. hypertension and atherosclerosis[5]. Since the morbidity and mortality of the diseases are high, the protection of the vascular endothelia has been a long-term focus of research. Multiple factors have been demonstrated to be capable of causing functional disorders of vascular endothelia, for instance, oxidative stress and inflammation which can lead to endothelial apoptosis, reduction of nitric oxide (NO) synthesis and increase of active substances (e.g. secretion of adhesion molecules)[6]. These pathophysiological changes of the endothelia not only elevate blood pressure but also alter the function and structure of the vascular tissues, and eventually give rise to vascular remodeling and hypertension[5, 7]. When the endothelia are injured and its morphology changes, the gap between the endothelial cells will be widened and monocytes be accumulated and foamed inside the vascular walls[5]. Then, hypertension and atherosclerosis will ensue. Studies have verified that oxidative stress and inflammation through injuring the endothelia play important roles in the process of the vascular disease development and that protection of the endothelia against the damage caused by the oxidative stress and inflammation is an important approach to treating the vascular diseases[6, 8, 9]. Therefore, finding active substances against the oxidative stress and inflammation has become a general and effective way to fight the vascular diseases.

The vascular endothelia have been shown to express TrkB receptors[10]. Therefore, it will be interesting to know whether or not 7, 8-DHF is an active substance for the endothelia being capable of acting on the TrkB receptors and activating any biological functions, such as anti-oxidative stress or anti-inflammation. In this study, we used a human umbilic vein endothelial cell (HuVEC) line, EA.hy926, as a cell model and applied *in vitro* treatment with H_2_O_2_ to cause cellular lesion, imitating *in vivo* status of oxidative stress and inflammation. Using the model, we found that 7, 8-DHF binding to TrkB receptors did prevent the cellular damage caused by H_2_O_2_ through suppression of apoptosis, attenuation of inflammatory factor releasing and inhibition of reactive oxygen species generation. The possible protective mechanisms were studied as well.

## Materials and Methods

### Materials

7, 8-dihydroxyflavone (TCI laboratories, Tokyo, Japan) was dissolved in dimethylsulfoxide (DMSO)to 100 mM as a stock solution. ANA-12 (Cat^#^ BTB06525, N2-(2-phenyl)benzo-thiophene-2-carboxamide) was purchased from Maybridge (Fisher Scientific Worldwide, Shanghai, China); H_2_O_2_ (hydrogen peroxide)and MTT (3-(4,5-dimethylthiazol-2-yl)-2,5-diphenyltetrazolium bromide) from Sigma-Aldrich (St. Louis, MO, USA); DAPI from Immunochemistry Technologies (Bloomington, USA); Antibodies against cytochrome C, Akt, phosphorylated Akt, NF-κB and IκB were purchased from Cell Signaling Technology (Danvers, MA, USA); antibody against HO-1 from Abcam (Cambridge, MA, USA); the anti-β-actin antibody was procured from Bioss (Beijing, China); goat anti-rabbit IgG-HRP antibody from Santa Cruz Biotechnology (Paso Robles, CA, USA);Goat anti-rabbit IgG antibody conjugated with FITC from Sigma-Aldrich (St. Louis, MO, USA).

### MTT assay

MTT assay was used to estimate the cell proliferation with the tetrazolium dye. Cells were seeded into a 96-well plate at a density of 1x10^4^ cells per well. Then the experimentally treated cells were incubated with 20 μL MTT solution (5 mg/mL in PBS) for 4 h at 37°C. The formazan crystals formed in the intact cells were dissolved in 150 μL DMSO. The absorbance was measured at 490 nm with a microplate reader (Molecular Devices, Sunnyvale, CA, USA).

### DAPI staining and flow cytometry for apoptotic determination

EA.hy926 cells were cultured on coverslips, treated with bolus addition of H_2_O_2_ or/and 7, 8-DHF for 24 hrs and stained with DAPI staining solution (Leagene Biotech, Beijing, China). The stained nuclei were observed under Leica DMI 4000B fluorescent microscope (Leica Microsystems, Wetzlar, Germany).

The treated EA.hy926 cells were digested with trypsin without EDTA added, collected and suspended in a binding buffer at a cell density of 1x10^6^/mL. Cells were mixed with an Annexin V-FITC/PI Apoptosis Detection Kit (Invitrogen/Life Technologies, Carlsbad, CA, USA) as instructed by the manufacturer, filtered with 200 mesh screens and passed through the flow cytometer(BD FACSCalibur, BD Biosciences, San Jose, USA) for apoptotic detection and analyses.

### Cell cultures, protein extraction and western blotting for cellular proteins

EA.hy926, a human umbilic vein endothelial cells (HuVECs)-derivedcell line (purchased from the Type Culture Collection of the Chinese Academy of Sciences, Shanghai, China)was cultured and passaged in DMEM media (Gibco, GrandIsland,USA) supplemented with 10% fetal bovine serum (Gibco). For total protein extraction, cells were homogenized in a lysis buffer (Beyotime Institute of Biotechnology, Shanghai, China)containing 50 mM Tris (pH 7.4), 150 mM NaCl, 1% Triton X-100, 1% sodium deoxycholate, 0.1% SDS, 2 mM sodium pyrophosphate, 25 mMβ-glycerophosphate, 1 mM EDTA, 1 mM Na_3_VO_4_, 0.5 μg/mL leupeptin, and 1 mM PMSF. PMSF was prepared immediately before use. The homogenates were centrifuged and the protein concentrations in the supernatants were determined as described[11]. SDS-PAGE for the supernatants, western blotting and chemiluminescence were performed as previously[11].

For cytoplasmic protein extraction, cells were washed in ice-cold PBS, centrifuged and resuspended in the ice-cold hypotonic buffer form a kit (Beyotime Institute of Biotechnology, Shanghai, China) and incubated on ice for 10 min, vortexed slightly and centrifuged at 15,000 g for 1 min at 4°C. The supernatant (cytoplasmic protein) was collected and stored at −80°C.

### Quantitative RT-PCR

Total RNA was extracted from EA.hy926 cells with Trizol reagent(Invitrogen/Life Technologies) according to the manufacturer’s protocol. Two micrograms of totalRNA was reverse-transcribed into complementary DNA. The levels of mRNA of interest were quantified by real-time RT-PCR using SYBRGreen Master Mix(Life Technology, Carlsbad, USA) with Light Cycler 480 II (Roche Applied Science, Penzberg, Upper Bavaria, Germany). The relative mRNA expression of interest genes was represented by2^-ΔΔCt^.ΔΔCt = average ΔCt_(treatment)_ − average ΔCt_(control)_; ΔCt = Ct_(gene of interest)_ − Ct_(GAPDH)_.

The specific primers designed and used in the study were:

TNFα: sense: 5’-TGCTTGTTCCTCAGCCTCTT-3’

antisense: 5’-CAGAGGGCTGATTAGAGAGAGGT-3’

IL-1β: sense: 5’-TGAAGCAGCCATGGCAGAAG-3’

antisense: 5’-GGTCGGAGATTCGTAGCTGGA-3’

ICAM1: sense: 5-TGTATGAACTGAGCAATGTGCAAGA-3’

antisense: 5’-TGTATGAACTGAGCAATGTGCAAGA-3’

GAPDH: sense: 5’-GCACCGTCAAGGCTGAGAAC-3’

antisense: 5’-TGGTGAAGACGCCAGTGGA-3’

The primers were synthesized by Takara (Dalian, China).

### Fluorescent immunocytochemistry

EA.hy926 cells were cultured on coverslips, treated with bolus addition of H_2_O_2_ or/and 7, 8-DHF for 24 hrs and stained with an antibody against NF-κB (1:1000) and a secondary FITC-conjugated antibody (1:200). Cellular fluorescence was observed under the Leica DMI 4000B fluorescent microscope.

### Measurement of cellular MDA and SOD activity

The commercial assay kits(Jiancheng Bioengineering Institute, Nanjing, China) were used for measurement of cellular malondialdehyde (MDA) content and cellular total superoxide dismutase(SOD) activity as described[3]. Briefly, EA.hy926 cells were homogenized and centrifuged for supernatants whose protein concentrations were then determined according to the instruction of the manufacturer before MDA or SOD assays were done. The unit for MDA content was nmol/mg protein and U/mg protein for SOD activity.

### Statistical analyses

Data were expressed as means ± SEM. Student’s*t* tests was used to compare the differences between means of two groups. One-way analysis of variance (ANOVA) was used when the means were compared among multiple groups. Statistical analyses were performed with Graph-Pad Prism 5.0 (GraphPad Software, La Jolla, CA, USA). A probability value of P < 0.05 was considered to be statistically significant.

## Results

### 1. 7, 8-DHF via TrkB receptors affected the viability of EA.hy926 cells exposed to H_2_O_2_

**Fig 1:**
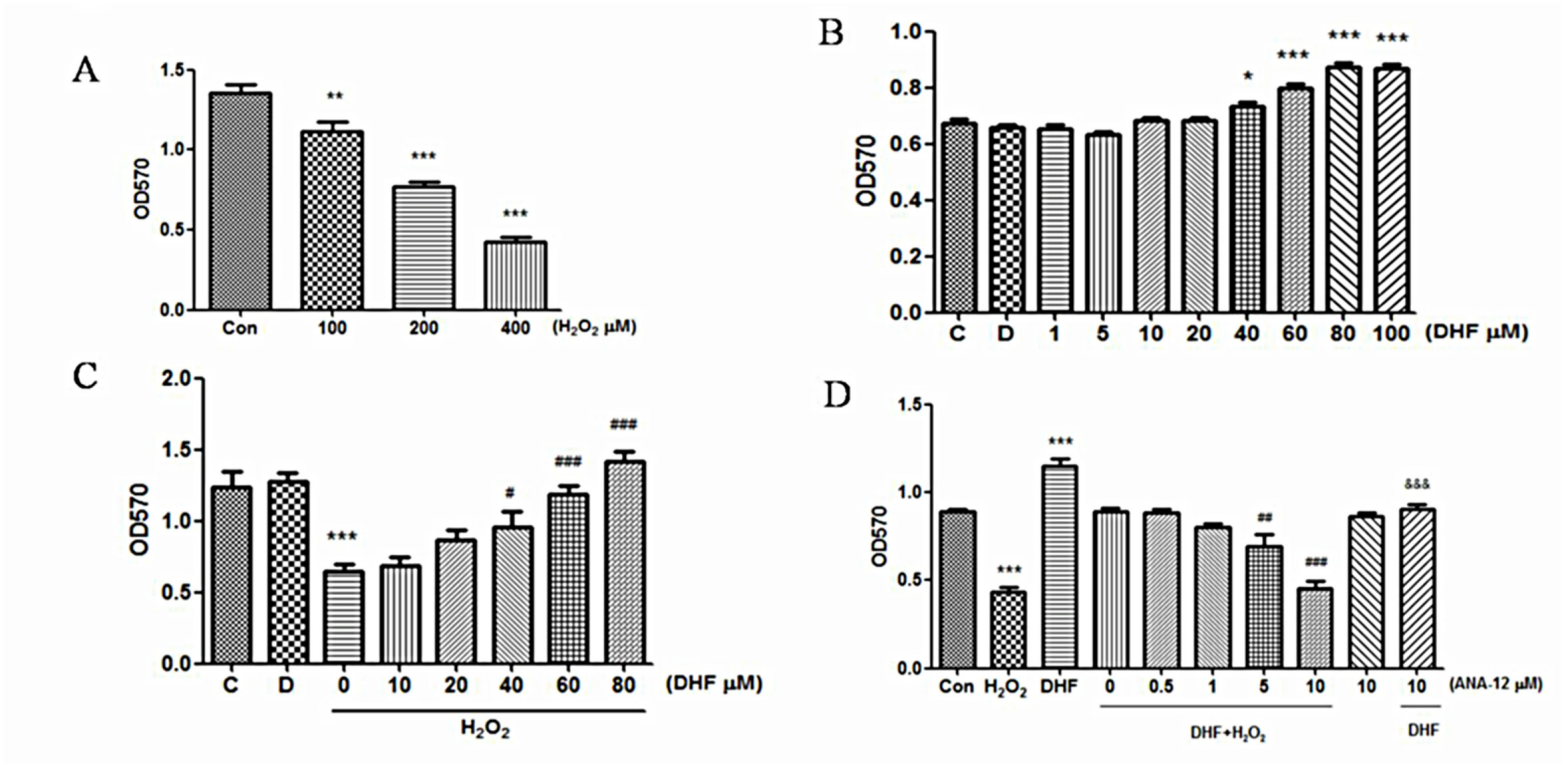
7, 8-DHF increased the viability of H_2_O_2_-treated EA.hy926 cells through TrkB receptors. Cell viability was examined with MTT assay. Drugs were added to cell medium in bolus. **(A)** Viability of EA.hy926 cells was significantly weakened by H_2_O_2_ dose-dependently. **(B)**Proliferation-enhancing effect of 7, 8-DHF on EA.hy926 cells. **(C)** 7, 8-DHF dose-dependently reversed the H_2_O_2_-damaged cellular viability. **(D)** TrkB antagonist AN1-12 blocked the protecting effect of 7, 8-DHF. *, ** or *** indicates P < 0.05, 0.01 or 0.001 for comparison between control (Con, C or D; D means addition of DMSO alone.) and H_2_O_2_, DHF or DHF+H_2_O_2_ treatment; #, ## or ### indicates P < 0.05, 0.01 or 0.001 for comparison between H_2_O_2_ alone and H_2_O_2_+DHF (C) or between H_2_O_2_+DHF and H_2_O_2_+DHF+ANA-12 (D); &&& indicates P < 0.001 for comparison between DHF alone and DHF+ANA-12 (D). *n* = 6 for A, B, C and D.

In order to observe whether there is any protecting effect of 7,8-DHF on endothelial cells, we selected EA.hy926 as a cell model and first examined the dose-dependent cellular damage by H_2_O_2_ with the method of MTT assay. Cells were incubated with different concentrations of H_2_O_2_ for 24 hours. The results showed that H_2_O_2_ inhibited the proliferation of EA.hy926 cells in a concentration-dependent manner, and the cell viability decreased to about 50% of the control at 200μM of H_2_O_2_ concentration (P <0.001, Fig 1A). H_2_O_2_, an endogenous ROS (reactive oxygen species), is produced intracellularly and exists steadily at physiological concentrations of 0.01–0.1μM[12]. However, the threshold value for the concentration of intracellular steady state H_2_O_2_ that induces apoptosis was estimated in Jurk at T cells to be around 0.7 μM[13]. The concentrations of H_2_O_2_ used to study the redox regulation in physiological processes in experimental cell models exposed to bolus addition of H_2_O_2_ were from 10 to 1000 μM[13]. Therefore, 200 μM of H_2_O_2_ is regarded as a reasonable concentration which can significantly and fully cause oxidative injury to the EA.hy926 cells. This concentration was selected for the following experiments.

Before examining whether 7, 8-DHF was able to prevent the H_2_O_2_-damaged EA.hy926 cells, we examined the effect of 7, 8-DHF on their proliferation. As shown in Fig 1B, low dose of 7,8-DHF had slight effect while high dose (40 μM or higher) significantly increased the proliferation. In order to decide which concentrations of 7, 8-DHF were able to efficiently affect cellular viability, H_2_O_2_ at 200 μM was applied to EA.hy926 cells pre-treated with 7, 8-DHF of serial concentrations. Consistent with the results in Fig 1B, the proliferation-enhancing effect of 7, 8-DHF at concentrations of 40 μM higher significantly inhibited the cell injury induced by H_2_O_2_ (Fig 1C).

Since TrkB receptors exist on the endothelial cells[10], we hypothesized that the protecting effect of 7, 8-DHF as a TrkB receptor agonist should have been initiated from the receptors. We firstly confirmed that the proliferation-enhancing effect of 7, 8-DHF was mediated by TrkB receptors because ANA-12[14], a TrkB receptor specific antagonist at 10 μM almost completely abrogated the cellular proliferation enhanced by 7, 8-DHF at 60 μM (Fig 1D, bars1, 3 and 10 from left)while ANA-12 alone at 10μM did not affect the proliferation(bars 1 and 9). Subsequently, using ANA-12, we demonstrated that the protecting role 7,8-DHF had played in EA.hy926 cells against H_2_O_2_ was blocked by the antagonist at 5 and 10μM (Fig 1D, bars 4, 7 and 8), implying that the protecting role of 7, 8-DHF against H_2_O_2_ injury was also mediated by the TrkB receptors.

### 2. 7, 8-DHF inhibited H_2_O_2_-induced apoptosis in EA.hy926 cells

**Fig 2:**
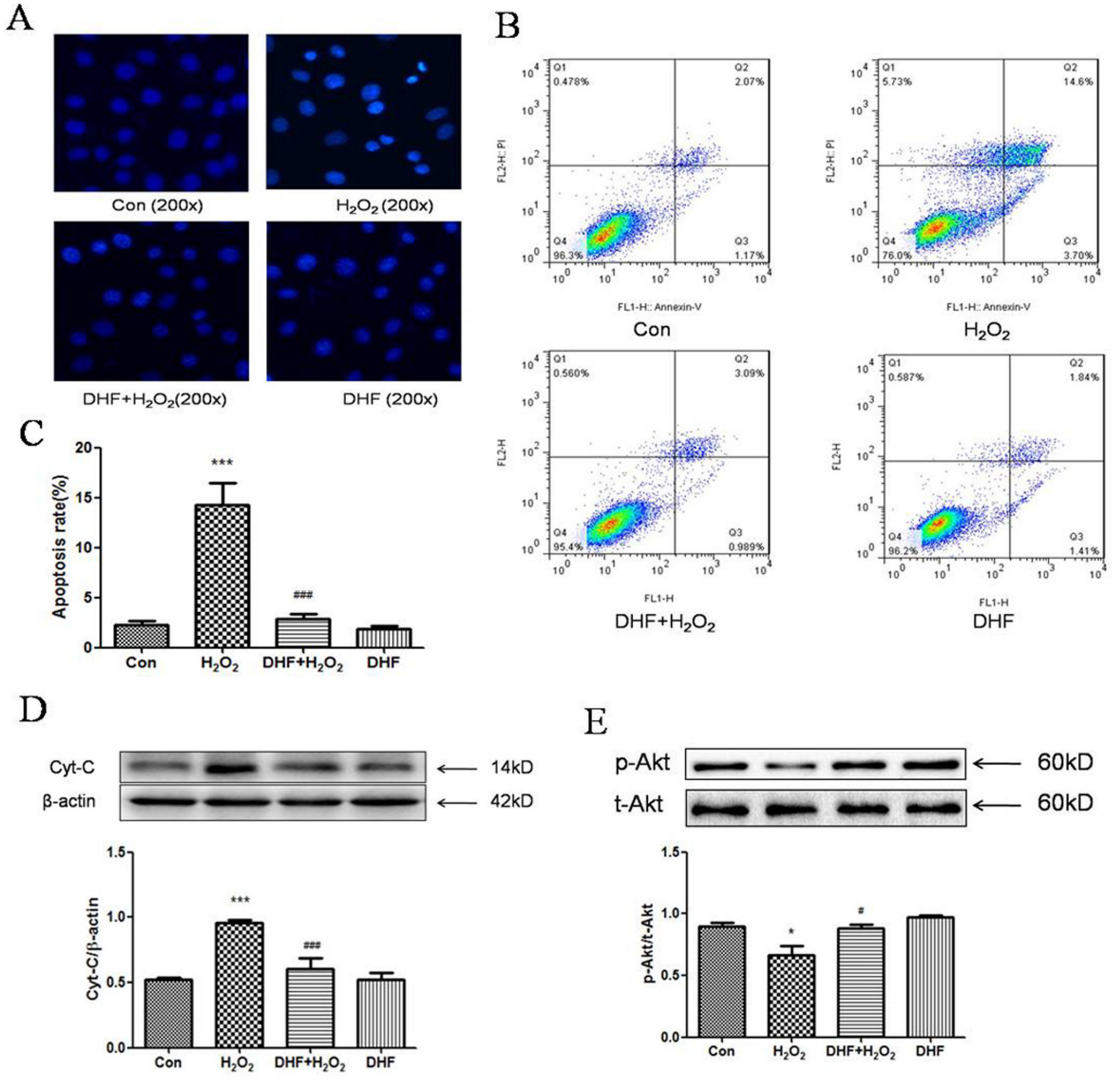
7, 8-DHF inhibited H_2_O_2_-induced apoptosis in EA.hy926 cells. **(A)** DAPI-stained nuclei of H_2_O_2_ or/and 7, 8-DHF-treated EA.hy926 cells were shown under fluorescent microscopy. 200x indicates magnification. **(B)** and **(C)** Flow cytometry for H_2_O_2_ or/and 7, 8-DHF-treated EA.hy926 cells shows the apoptotic rates of one representative experiment (B) and the average rates (C). **(D)** and **(E)** Western blottings show the changes of cytochrome C (D), total Akt (t-Akt) and phosphorylated Akt (p-Akt) (E) protein levels after H_2_O_2_ or/and 7, 8-DHF treatments. * or *** indicates P < 0.05 or 0.001 for comparison between control (Con) and H_2_O_2_ treatment; # or ### indicates P <0.05 or 0.001 for comparison between H_2_O_2_ and H_2_O_2_+DHF treatment. *n* = 4 for A, 6 for B, and 4–6 for D and E.

**Fig 3:**
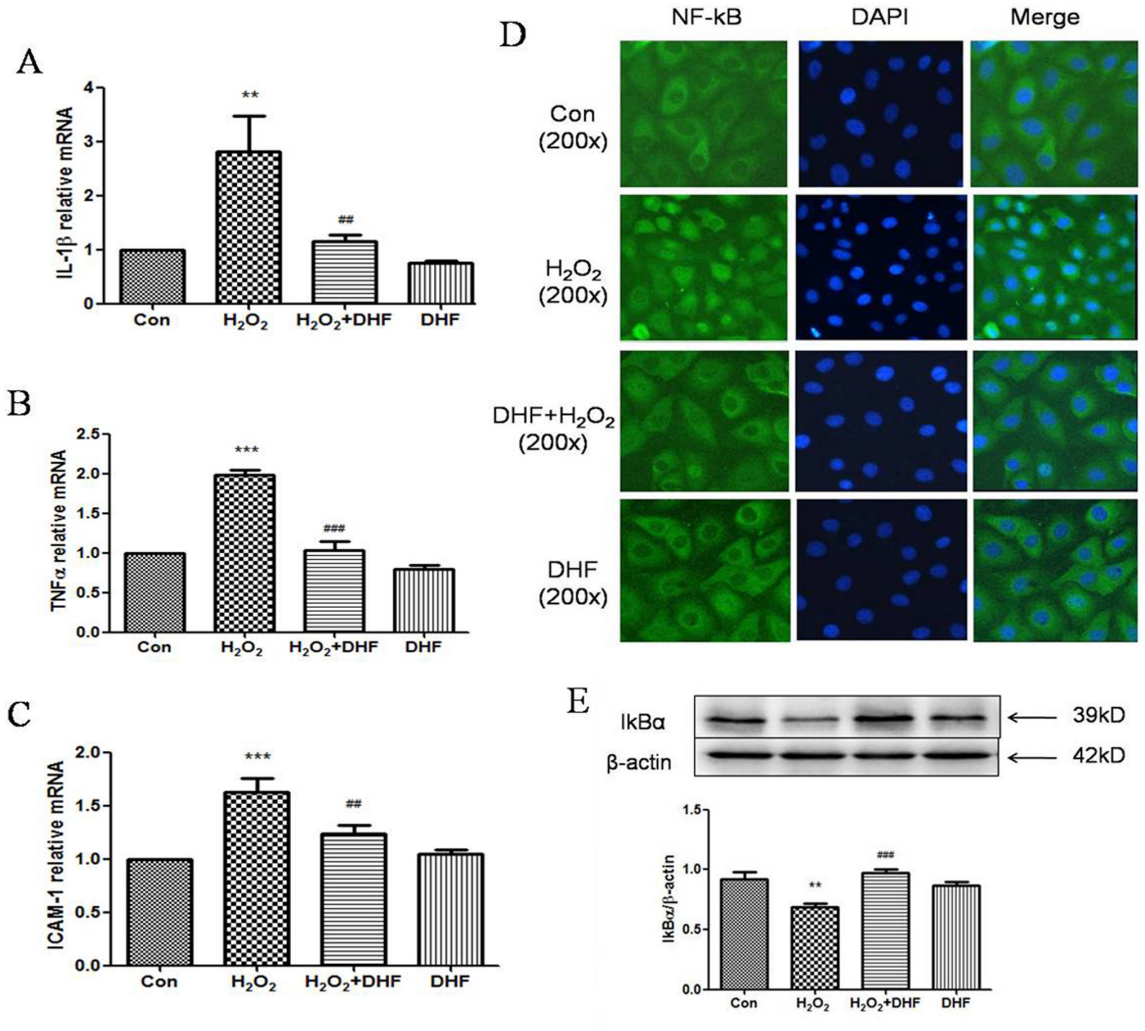
7, 8-DHF attenuated H_2_O_2_-induced inflammation in EA.hy926 cells. **(A), (B)** and **(C)** Quantitative RT-PCRs show the changes of IL-1β (A), ICAM-1 (B) and TNFα (C) mRNA relative levels (Y-axis, see Methods for details) after H_2_O_2_ or/and 7, 8-DHF treatments. **(D)** Fluorescent immunocytochemistry shows the nuclear translocation of NF-κB in cells treated with H_2_O_2_ under fluorescent microscopy with 200x magnification. Blue color indicates DAPI staining of nuclei; Green the staining of NF-κB recognized by antibodies. The secondary antibody was conjugated with FITC. **(E)** Western blotting shows the changes of cytoplasmic IκBα protein levels after H_2_O_2_ or/and 7, 8-DHF treatments. ** or *** indicates P < 0.01 or 0.001 for comparison between control (Con) and H_2_O_2_ treatment; ## or ### indicates P < 0.01 or 0.001 for comparison between H_2_O_2_ and H_2_O_2_+DHF treatment. *n* = 4 for A, B and C, 6 for D, and 4 for E.

It is well known that H_2_O_2_ reduces cellular viability through apoptosis[13]. We assumed, therefore, that 7, 8-DHF protected H_2_O_2_–attacked EA.hy926 cells by way of apoptotic suppression. The effect of 7,8-DHF on apoptosis induced by H_2_O_2_ was studied with DAPI staining and flow cytometry. The DAPI staining showed that the number of apoptotic cells induced by H_2_O_2_ was significantly increased compared with the control group while 7, 8-DHF evidently reduced the apoptotic number (Fig 2A). The nuclear morphology showed typical apoptotic characters: nuclear condensation and fragmentation (Fig 2A) and the size of the H_2_O_2_-treated cells was also lessened obviously (Fig 3D). Using flow cytometry to quantitatively observe the apoptotic rate of EA.hy926 cells, compared with the control group (4.01±0.43%), we found that the apoptotic rate of H_2_O_2_-treated EA.hy926 cells was significantly increased to 14.23±2.22% by about 3.5-fold. However, treatment with 7, 8-DHF lowered this rate back to 5.68± 0.52%(P<0.001, Fig 2B and 2C).

In the subsequent experiments, we examined the changes of two molecular markers of apoptosis, cytochrome C and Akt[15–17]. H_2_O_2_-induced apoptosis was usually accompanied by cytochrome C releasing from mitochondria and H_2_O_2_ treatment inhibited phosphorylation of Akt[15–17]. As expected, western blotting showed that H_2_O_2_ increased the protein level of cytoplasmic cytochrome C in EA.hy926 cells compared with the control group (P<0.001) while 7, 8-DHF significantly reduced the protein level of cytoplasmic cytochrome C (P<0.001, Fig 2D). Simultaneously, the phosphorylation level of Akt was found to have decreased significantly in the H_2_O_2_ group while 7, 8-DHF significantly enhanced the phosphorylation of Akt in EA.hy926 cells in the presence or absence of H_2_O_2_ (Fig 2E). The changes of apoptotic markers supported that H_2_O_2_ initiated the apoptotic process but 7, 8-DHF prevented the process in the H_2_O_2_-treated EA.hy926 cells.

### 3. 7, 8-DHF attenuated H_2_O_2_-induced inflammation in EA.hy926 cells

Another factor affecting cellular viability is cell inflammation. Using quantitative real-time PCR, the effect of 7,8-DHF was investigated on the H_2_O_2_-induced mRNA expression in EA.hy926 cells of three major inflammatory factors, IL-1β, ICAM-1 and TNFα[18]. Results showed that H_2_O_2_ significantly stimulated the expression of IL-1β, ICAM-1 and TNFα mRNAs compared with control EA.hy926 cells while 7,8-DHF pretreatment significantly suppressed the expression of these genes in the presence of H_2_O_2_ (Fig 3A, 3B and 3C), indicating that 7, 8-DHF may have attenuated H_2_O_2_-induced inflammation in EA.hy926 cells.

It is known that NF-κB is involved in regulating the transcription of many inflammatory genes in vascular inflammation[19, 20]. NF-κB is activated through IκB degradation in the process of nuclear translocation[21]. To explore the mechanism by which 7,8-DHF suppressed inflammation induced by H_2_O_2_, NF-κB nuclear translocation induced by H_2_O_2_ in EA.hy926 cells was investigated. The cellular localization of the p65, a subunit of NF-κB, was examined by fluorescent immunocytochemistry. As shown in Fig 3D, in the control cells (Con), NF-κB was localized predominantly in cytoplasm. However, H_2_O_2_ treatment significantly induced NF-κB to transfer from the cytoplasm into the nuclei while 7,8-DHF pretreatment significantly blocked the nuclear translocation. 7,8-DHF alone did not affect the nuclear translocation apparently.

IκB is one of determinant proteins for NF-κB nuclear translocation and its degradation plays an important role to activate the process[21]. Consistently, when EA.hy926 cells were treated with H_2_O_2_ and the NF-κB nuclear translocation occurred, the decreased protein level of IκB in the cytoplasm was detected by western blotting (P < 0.001, Fig 3E). Co-treatment with both 7,8-DHF and H_2_O_2_ prevented the decrease of IκB protein expression. The results indicated that the expression of the inflammatory factors stimulated/suppressed by H_2_O_2_/7,8-DHF was mediated by NF-κB and IκB factors.

### 4. 7, 8-DHF prevented generation of ROS induced by H_2_O_2_ in EA.hy926 cells

**Fig 4:**
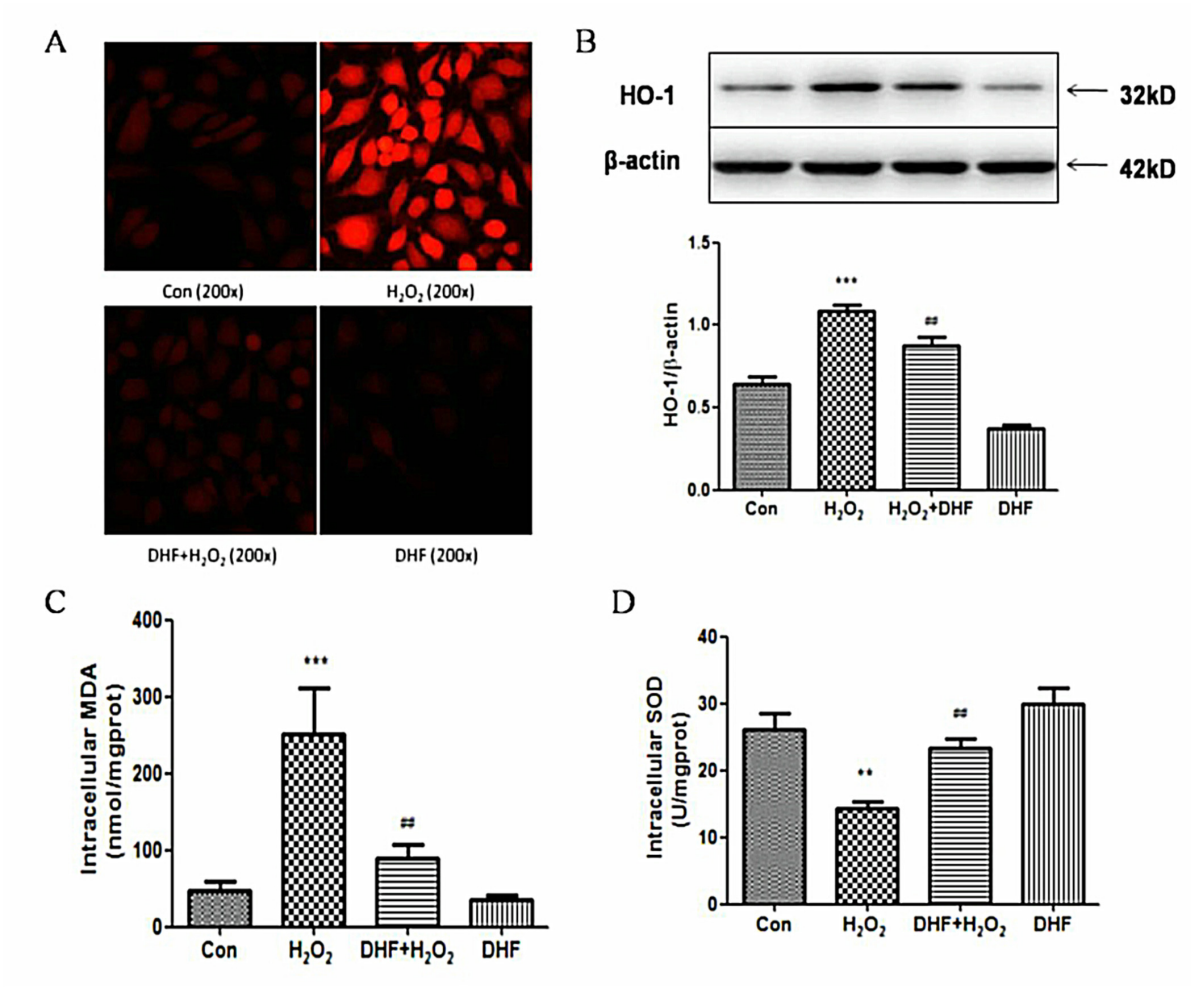
7, 8-DHF prevented generation of ROS and suppressed responses to oxidative stress induced by H_2_O_2_ in EA.hy926 cells. **(A)** ROS staining of cells treated with H_2_O_2_ or/and 7, 8-DHF detected under fluorescent microscopy (200x magnification). **(B)** Western blotting to detect the changes of HO-1 protein amount expressed in cells treated with H_2_O_2_ or/and 7, 8-DHF. **(C)** and **(D)** Measurement of cellular MDA amount and SOD activity. With assay kits (Methods), intracellular MDA amount (C) and SOD activity (D) were measured for the cells treated with H_2_O_2_ or/and 7, 8-DHF. ** or *** indicates P < 0.01 or 0.001 for comparison between control (Con) and H_2_O_2_ treatment; ## indicates P < 0.01 for comparison between H_2_O_2_ and H_2_O_2_+DHF treatment. *n* = 6 for A, B, C and D.

We have evidenced that 7, 8-DHF possesses antioxidant properties in PC12 cells[3, 4]. To investigate if 7, 8-DHF plays an antioxidant role in EA.hy926 cells, ROS fluorescence density was observed in the cells by fluorescent immunocytochemistry. As shown in Fig 4A, H_2_O_2_ could significantly increase the fluorescent intensity of cytoplasm compared with control while the H_2_O_2_–induced ROS fluorescence intensity was drastically weakened in the EA.hy926 cells pretreated with 7,8-DHF.

Subsequently, we studied three factors related with cellular oxidative stress, HO-1 (heme oxygenase-1), MDA and SOD. HO-1 and SOD are important intracellular antioxidants[22–24]. MDA is a product of lipid oxidation[25]. The changes of their intracellular levels reflect the state of oxidative stress in cells. The protein expression of HO-1 and intracellular MDA level are kept in relatively low levels in resting EA.hy926 cells. However, they were significantly increased by H_2_O_2_ treatment while 7, 8-DHF pretreatment prevented both the H_2_O_2_–induced increase of HO-1 protein expression and the elevated level of intracellular MDA in EA.hy926 cells (P< 0.001, Fig 4B and 4C). 7,8-DHF pretreatment could significantly enhance the cellular SOD activity of EA.hy926 cells, which was severely inhibited by H_2_O_2_ (P <0.001, Fig 4D). The results indicated that 7, 8-DHF had a significant antioxidant effect and its antioxidant property was associated with the activities of cellular HO-1 and SOD.

## Discussion

### Biological functions of 7, 8-DHF

The biological roles or functions of 7, 8-DHF are being investigated actively at present. Nonetheless, the focus of the researches has fallen on its biological effects on the neuro-regeneration and neuro-protection[26–28]. This is ascribed to the fact that 7, 8-DHF has been demonstrated to be a selective and hydrophobic small molecule agonist for TrkB receptors which are predominantly localized to central nervous system and play important roles in neurotrophy[1]. 7, 8-DHF is very promising to be a potent substitute for natural agonist of the receptors, brain-derived neurotrophic factor(BDNF). Since the TrkB receptors are also distributed in the peripheral tissues, such as vascular endothelia[10], we thought that the roles of 7, 8-DHF in the functions of the tissues may also evident and therefore, studied its effects on the vascular contraction[2]. To be continued, its effect on the endothelial survival from oxidative-stress injury had been investigated in this project. We found that 7, 8-DHF was potent to protect the endothelial cell lineEA.hy926from injury caused by H_2_O_2_ exposure.

### How did 7, 8-DHF protect EA.hy926 cells from the damage caused by H_2_O_2_?

H_2_O_2_ is an endogenous ROS and has been long recognized as a destructive molecule whose oxidative effect on biological activities of cells has been well studied[29]. H_2_O_2_ induces endothelial apoptosis by promoting the release of cytochrome C from mitochondria and starting mitochondrial apoptosis pathway, and the activated Akt can inhibit the release of cytochrome C[15–17]. We had investigated the antioxidant action of 7, 8-DHF against the 6-hydroxydopamine-induced cytotoxicity in PC-12 cells and found that it protected the cells through PI3K/Akt and JNK pathways[3, 4]. Since there seemed to be no TrkB receptors on PC-12, it was thought that 7, 8-DHF diffused into cytoplasm through plasma membrane and acted directly on the pathway factors to produce the antioxidant effects.

TrkB receptors have been demonstrated to be expressed on the endothelial cells[10]. Therefore, 7, 8-DHF was supposed to play an antioxidant roles in EA.hy926 cells through binding the receptors. The experiment with TrkB receptor-specific antagonist ANA-12 clarified this point of view (Fig 1D). Activation of the TrkB receptors by 7, 8-DHF triggered the signaling of PI3K/Akt pathway in neurons[1], and this pathway was also activated by 7, 8-DHF in EA.hy926 cells (Fig 2E), supporting that the TrkB receptors was activated by 7, 8-DHF, indeed.

Our previous and current experiments about the antioxidant effect of 7, 8-DHF in different types of cells (EA.hy926 and PC12) have brought forward an interesting aspect in the biological functions of the compound. TrkB receptors exist on EA.hy926 cells[30]. Therefore, it is intuitionistic to understand that the antioxidant effect of 7, 8-DHF in EA.hy926 cells is mediated via TrkB receptors. However, the receptors were not supposed to exist on PC12 cells but 7, 8-DHF played an antioxidant role in PC12 cells as well[3]. Since 7, 8-DHF is hydrophobic and membrane permeable, it is able to enter cells for its biological functions. To our limited knowledge, it remains unknown at present how 7, 8-DHF plays any biological roles inside cells. Nonetheless, it is noteworthy that 7, 8-DHF even at 5 μM effectively protected PC12 cells against 6-Hydroxydopamine (6-OHDA)-induced cell death (Fig 1B in reference 3). The toxic effect of 6-OHDA has been linked to overproduced cellular ROS such as H2O2. These results suggest that the antioxidant potency and mechanism of 7, 8-DHF probably are different among cell types. The mechanism for intracellular antioxidant influence of 7, 8-DHF requires further exploration.

In this study, we have observed that 7, 8-DHF was able to change the cellular levels of cytochrome C, HO-1, SOD activity, Akt phosphorylation, IκB degradation and NF-κB nuclear translocation. However, how these protein factors were stimulated or suppressed by 7, 8-DHF through TrkB receptors remained to be elucidated.

### The significance that 7,8-DHF protects vascular endothelia

The *in vitro* effect of 7, 8-DHF on the damage of vascular endothelia has implied its practicability in health care. ROS is being produced constantly from cells inside the body and released into the blood. Vascular endothelia are supposed to be the forefront of oxidative defense. The oxidative stress causes cellular apoptosis and inflammation, etc.[31]. Certainly, if the oxidative stress cannot be released as soon as possible, the endothelia will be destined to be damaged.

We have evidenced that 7, 8-DHF is a potent antioxidant compound and can alleviate the oxidative state, lower the apoptotic rate and attenuate the inflammation induced by H_2_O_2_. If the concentration of 7, 8-DHF in blood serum can be elevated to some extent, we believe that it will become a promising health products for improving the health of cardiovascular system. For instance, in spontaneously hypertensive rats (an animal model for study of human essential hypertension), a large number of ROS accumulation in the vascular tissue induced inflammation of vascular endothelial cells, leading to endothelial dysfunction[32]. We hope that application of 7, 8-DHF will alleviate the inflammation of vascular endothelia in hypertensive animals or humans. Another example was that 7, 8-DHF could inhibit the secretion of inflammatory cytokines from BV2 mouse microglia induced by LPS through suppressing the NF-κB and MAPK signaling pathways[33]. Our study showed similar results. Therefore, development of 7, 8-DHF into a health product will definitely benefit endothelial function.

In conclusion, 7, 8-dihydroxyflavone (7, 8-DHF), a selective agonist for TrkB receptors with strong neurotrophic functions, also protected the EA.hy926 cells, a human umbilic vein endothelial cell line which was exposed to hydrogen peroxide (H_2_O_2_), through suppression of apoptosis, attenuation of inflammatory factor releasing and inhibition of reactive oxygen species generation. The role in the EA.hy926 cells was mediated by its binding to TrkB receptors.
